# Turbulent particle pair diffusion: A theory based on local and non-local diffusional processes

**DOI:** 10.1371/journal.pone.0202940

**Published:** 2018-10-03

**Authors:** Nadeem A. Malik

**Affiliations:** Department of Mathematics and Statistics, King Fahd University of Petroleum and Minerals, P.O. Box 5046, Dhahran 31261, Saudi Arabia; Newcastle University, UNITED KINGDOM

## Abstract

A re-appraisal of the Richardson’s 1926 dataset [Richardson, L. F. Proc. Roy. Soc. Lond. A 100, 709–737, (1926)] displays an unequivocal non-local scaling for the pair diffusion coefficient, $K∼\sigma_{l}^{1.564}$, quite different to the previously assumed locality scaling law $∼\sigma_{l}^{4/3}$, where *σ_l_* is the pair separation. Consequently, the foundations of turbulent pair diffusion theory are re-examined here and it is shown that pair diffusion is governed by both local and non-local diffusional processess inside the inertial subrange. In the context of generalised energy spectra, *E*(*k*) ∼ *k*^−*p*^ for 1 < *p* ≤ 3, the new theory predicts *two* non-Richardson regimes depending on the size of the inertial subrange: (1) in the limit of asymptotically infinite subrange, non-local scaling laws is obtained, $K∼\sigma_{l}^{\gamma }$, with *γ* intermediate between the purely local and the purely non-local scalings, i.e. (1 + *p*)/2 < *γ* ≤ 2; and (2) for finite (short) inertial subrange, local scaling laws are obtained, $K∼\sigma_{l}^{(1+p)/2}$. The theory features a novel mathematical approach expressing the pair diffusion coefficient through a Fourier integral decomposition.

## 1 Introduction

Turbulent transport and mixing play an essential role in many natural and industrial processes [1], in cloud formation [2], in chemically reacting flows [3] and combustion systems [4, 5], and atmospheric and oceanographic turbulence determines the spread of pollutants and biological agents in geophysical flows [6–9]. Turbulent concentration fluctuations often play a critical role in such systems, and this is related to the separation of nearby fluid particles.

Turbulent particle pair diffusion (or relative diffusion) was introduced by L. F. Richardson [10], who laid the foundations for a theory of how ensembles of pairs of fluid particles (tracers) initially close together move apart due to the effects of atmospheric winds and turbulence. Pair diffusion is usually classified into three regimes depending on the pair separation distance *l* relative to the Kolmogorov length scale *η* of the turbulence: (i) the dissipation subrange where *l* ≪ *η*; (ii) the inertial subrange when *η* ≪ *l* ≪ *L*, where *L* is some outer length scale of the turbulence (such as the integral length scale or the Taylor microscale); (iii) at much larger times when *l* ≫ *L*, their motions become independent and the pair diffusion collapses to twice the one-particle Taylor diffusion, 〈*l*^2^〉 → 2〈*x*^2^〉 ∼ *t* [11].

Batchelor introduced a fourth regime, the so-called ballistic regime [12]. He noted that turbulence is correlated in time and space, so for very short times after release the motion should be well correlated with its initial conditions, thus the relative velocity is approximately constant for a short time which leads to $\langle l^{2}\rangle ∼l_{0}^{2}+v_{0}^{2}{\left(t-t_{0}\right)}^{2}$, where *v*_0_ = *v*(*t* = *t*_0_) is the rms pair velocity at initial time *t* = *t*_0_. In fact, this is true in any spatio-temporally correlated velocity field and not just in turbulence.

In this work, we focus our interest on inertial subrange scaling laws, and we will ignore the other regimes which are fairly well understood at the present time.

Richardson argued that as particle pairs separate in a field of turbulence, the rate at which they move apart is affected by different scales of motions, and the energies in the scales of motion (eddies) of the same scale as the pair separation are the most effective in the diffusional process—this is the so-called locality hypothesis. Most theories of turbulent pair diffusion since then have assumed locality.

Richardson was also motivated by a desire to bring molecular and turbulent pair diffusional processes into a unified picture through the use of a non-Fickian diffusion equation with scale dependent diffusion coefficient, *K*(*s*). Assuming homogeneous isotropic turbulence, Richardson posed the problem in 3D in terms of the probability density function (pdf) of the pair separation, *q* = *q*(*s*, *t*), subject to the normalization, $\int_{0}^{\infty }4\pi s^{2}q\left(s,t\right)ds=1$, he suggested the following diffusion equation to describe *q*,

$$\begin{array}{c} \frac{\partial q}{\partial t}=\frac{1}{s^{2}}\frac{\partial }{\partial s}(s^{2}K\left(s\right)\frac{\partial q}{\partial s}). \end{array}$$ (1)

Here, *s* is the sample space variable for the random pair separation distance. *K*(*s*) is also called the pair diffusion coefficient, or the pair diffusivity.

From observational data of turbulent pair diffusion coefficients collected from different sources, Richardson assumed an approximate constant power law fit to the data, *K*(*l*) ∼ *l*^4/3^. This is equivalent to [12, 13]

$$\begin{array}{c} \left\langle l^{2}\right\rangle ∼t^{3} \end{array}$$ (2)

often referred to as the Richardson-Obukov *t*^3^-regime. *l*(*t*) is the pair separation, at time *t*, and the angled brackets is the ensemble average over particle pairs.

It is no longer believed that it is possible to unify molecular and turbulent diffusional processes because their physics are fundamentally different; Brownian motion characterizes molecular diffusion, while convective gusts of winds that increase the pair separation in surges characterizes turbulent diffusion [14–16]; but the idea of a scale dependent turbulent diffusivity has survived.

Richardson’s assumed 4/3-scaling law is consistent with Kolmogorov turbulence K41 [17] in the following manner. The K41 energy spectrum in the inertial subrange is, *E*(*l*) ∼ *ε*^2/3^ *l*^5/3^, from which it follows that the pair diffusion coefficient depends only upon *l* and *ε* (the rate of kinetic energy dissipation per unit mass). The typical pair relative velocity in eddy scales of order *l* is $v\left(l\right)∼\sqrt{E(l)/l}$, and from the scaling $K\left(l\right)∼lv\left(l\right)∼\sqrt{lE(l)}$, we obtain directly the 4/3-scaling, *K*(*l*) ∼ *ε*^1/3^ *l*^4/3^.

This is remarkable because Richardson effectively anticipated K41 fifteen years ahead of Kolomogorov. It is usual to evaluate *K* at typical values of, *l*, namely at $\sigma_{l}=\sqrt{\langle l^{2}\rangle }$, so this scaling is replaced by, $K\left(l\right)∼\varepsilon^{1/3}\sigma_{l}^{4/3}$. We will follow the this convention in the present work.

The admittance of a solution to Eq (1) from a point source implies that the initial separation of paricle pairs is effectively zero, and therefore the Kolmogorov scale must also asymptote to zero. Richardson’s theory is thus true strictly only in the asymptotic limit of infinite Reynolds number, *Re* → ∞ (which is equivalent to infinite inertial subrange, *R_k_* → ∞), and also *l*_0_ → 0, as *t* → 0.

With the 4/3-scaling for *K*, an explicit self-similar solution of Eq (1) exists for diffusion from a point source with boundary conditions *q*(0, *t*) = *q*(∞, *t*) = 0,

$$\begin{array}{c} q\left(r,t\right)=\frac{429}{70}\sqrt{\frac{143}{2}}{\left(\frac{1}{\pi \langle l^{2}\left(t\right)\rangle }\right)}^{3/2}\text{exp}\left(-{\left(\frac{1287}{8\langle s^{2}\left(t\right)\rangle }\right)}^{1/3}\right). \end{array}$$ (3)

Turbulence is both scale dependent and time correlated (non-Markovian), and it is not clear whether the pdf in Eq (3), which describes a local and Markovian process, can accurately represent the turbulent pair diffusion process. However, attempts have been made to derive alternative non-Markovian models for pair diffusion, such as Levy Flight type models, [18–23], and this remains a topic of active research in the field.

It is possible to generalize the scaling for the diffusion coefficient to be time dependent and still be consistent with K41 and with locality, [24–26] such that,

$$\begin{array}{c} K∼\varepsilon^{a}t^{b}l^{c} \end{array}$$ (4)

for some *a*, *b*, and *c*. Dimensional consistency then gives, 2*a* + *c* = 2 and 3*a* − *b* = 1, which leads to *a* = 1/3 − *b*/3, and *c* = 4/3 − 2*b*/3. Thus we obtain,

$$\begin{array}{c} K∼\varepsilon^{\left(1/3-b/3\right)}t^{b}l^{\left(4/3-2b/3\right)} \end{array}$$ (5)

If the further constraint 2*b* + 3*c* = 4 is satisfied, then this yields, 〈*l*^2^〉 ∼ *t*^3^. Thus, a *t*^3^-regime is not a unique signature for 4/3-scaling. Indeed, Batchelor [12] proposed an explicit time-dependent diffusivity *K*(*t*) ∼ *εt*^2^. Richardson’s scaling corresponds to *b* = 0 in Eq (4), yielding *a* = 1/3 and *c* = 4/3, and Batchelor’s scaling corresponds to *c* = 0, yieding *a* = 1 and *b* = 2. Both give 〈*l*^2^〉 ∼ *t*^3^.

However, a time dependent diffussion coefficient is hard to justify physically if we assume steady state equilibrium turbulence, because it implies unrealistic values for *K* as time progresses: for *b* > 0 the pair diffusivity increases in time without limit; for *b* < 0 the pair diffusivity approaches zero in time and the separation process effectively stops. Both cases seem unlikely, and in the ensuing we will restrict the discussion to steady state equilibrium turbulence and consider only the case, *b* = 0.

It is worth remarking, however, that time-dependent diffusivities like Eq (4) may be appropriate in the context of non-equilibrium turbulence. Furthermore, Hentchel & Procaccia [26] discuss time-dependent diffusivity it the context of clouds of particles; in this case, we are not dealing with point-sources and the description of the pair diffusion by a diffusion-type equation like Eq (1) is questionable.

Some of the questions that we address in this work are as follows. What evidence is there for the locality hypothesis? Can we develop a theory for turbulent pair diffusion without introducing the assumption of locality from the beginning? What scalings laws for the pair diffusion coefficients does this yield, firstly in the limit of infinite inertial subranges (infinite Reynolds number), and secondly for finite inertial subranges (moderate Reynolds numbers)?

Here, we construct a new theoretical framework through a novel method of analysis namely the Fourier decomposition of the relative pair velocity. We derive the scaling laws for the pair diffusion coefficient without making the assumption of locality from the outset.

In [27], we investigate this new theory through numerical simulations and where all predictions of the theory presented here have been verified.

The rest of the paper is organised as follows. In Section 2 we re-examine the body of evidence available on turbulent pair diffusion especially from large scale turbulence containing large inertial subranges. In Section 3 we focuss upon a reappraisal of Richardson’s original 1926 dataset. In Section 4, a new theory that does not *a priori* make the assumption of locality is constructed through a novel mathematical method from which new scaling laws for the pair diffusivity is derived. In Section 5 some precitions from the new theory is derived. We discuss the implications of the theory in Section 6.

## 2 What is the evidence for locality?

The general consensus among scientists in the field at the current time is that the collection of observational data, experimental data, and Direct Numerical Simulation, suggests a convergence towards a Richard-Obukov locality scaling. However, the relatively low Reynolds numbers in the experiments and DNS, and the high error levels in collecting the data means that this is by no means a forgone conclusion. As noted by Salazar and Collins [25], “‥ there has not been an experiment that has unequivocally confirmed R-O scaling over a broad-enough range of time and with sufficient accuracy.” It is not known what size of the inertial subrange is required to observe unequivocally the pair diffusion scaling, but it is widely assumed to be many orders of magnitude. Only geophysical turbulence, such as in the atmosphere and in the oceans, can produce such extended inertial subranges. We define the size of the inertial subrange *R*_*k*_ to be,

$$\begin{array}{c} R_{k}=\frac{k_{\eta }}{k_{1}} \end{array}$$ (6)

where the inertial subrange part of the energy spectrum *E*(*k*) is defined in the wavenumber range *k*_1_ ≤ *k* ≤ *k_η_*.

Observations of approximate R-O *t*^3^-regimes in geophysical flows, have been reported by Tartarski [28], Wilkins [14], Sullivan [29], and Morel & Larchaveque [30]. More recent observations include Julian et al. [31] in the atmosphere, and Lacasce & Ohlmann [32], and Ollitrault & Gabillet [33] in the oceans. But the high error levels and other assumptions, such as two-dimensionality, made in these observations means that a firm conclusions cannot be drawn from them regarding pair diffusion theories.

Cases in point are the recent experiments in the atmosphere [34] and in the Nordic sea [35]. In [34], the authors revisited The EOLE experiment in 1973 to study turbulent processes in the lower stratosphere circulation Relative dispersion of balloon pairs was studied by calculating the finite-scale Lyapunov exponent, an exit-time-based technique. The improved analysis supports a *k*^−5/3^ behavior in the mesoscale range 100–1000 km. However, they were unable to deduce the origin of this spectrum—whether it concerns 2D inverse energy cascade, gravity wave breaking with direct energy cascade, or shear (zonal) dispersion in a diffusive (meridional) field.

In [35], the authors examine the relative dispersion of surface drifters deployed in the POLEWARD experiment in the Nordic Seas during 2007–2008. The authors found some evidence for Richardson pair diffusion but could not rule out that this may be due an inverse energy cascade as this is a quasi-2D system. The deformation circle when shear effects become important is small in the Nordic sea, so the Richardson diffusion was observed only in a short range over one decade of scales, 10–100 km, in separation distance.

Direct Numerical Simulations (DNS) is inconclusive at the current time because it does not produce a big enough inertial subrange in order to test pair diffusion laws convincingly. For example, Ishihara et al. [36] perform a DNS with 4096^3^, at Taylor scale based Reynolds numbers *R*_λ_ ≈ 1200 showing an approximate inertial subrange energy spectrum over a very short range of just 40. Other DNS of particle pair studies at low Reynolds numbers are, Yeung [37] at *R*_λ_ = 90, Boffeta & Sokolov [38] at *R*_λ_ = 200, Ishihara & Kaneda [39] at *R*_λ_ = 283, Yeung& Borgas [40] at *R*_λ_ = 230, Sawford et al. [41] at *R*_λ_ = 650. Scatamacchia et al. [42] at *R*_λ_ = 300. See also Bitane et al. [43], and Biferale et al. [44]. The maximum separation of time scales between the integral time scale and the Kolmogorov times scale observed to date in DNS is about a factor of, 100. This is still too small to fully test inertial subrange pair diffusion scalings. For 2D turbulence, see [45, 46].

Particle Tracking Velocimetry (PTV) laboratory experiments Mass et al. [47], and Malik et al. [48] have been providing pair diffusion statistics at low to moderate Reynolds numbers. Like DNS, these Reynolds numbers are too small to reliably test pair diffusion laws. Virant & Dracos [16] obtain pair diffusion measurements from PTV. More recently, Berg et al. [49] obtained measurements in a water tank at *R*_λ_ = 172, and Bourgoin et al. [50] and Ouellete et al. [51] tracked hundreds of particles at high temporal resolution at *R*_λ_ = 815. Although higher resolution tracking experiments using high-energy physics methods have been performed for single particle trajectories [52], they have not yet been applied to particle pair studies.

Apart from experiments and DNS, many models of turbulent relative particle diffusion have been proposed from classical stochastic random flight type models [53, 54], to more recent multifractal models of turbulence [55]. However, none of these models address the problem of local and non-local diffusional processes directly, and as such it is not possible to compare them with the new model developed here. We will therefore not refer to such models any further in this study.

Datasets, both numerical and experimental, on relative dispersion are being made available on a number of databases such as [56] and [57].

In summary, at the current time the scaling laws for inertial subrange pair diffusion remain inconclusive.

## 3 A reappraisal of the 1926 dataset

**Fig 1:**
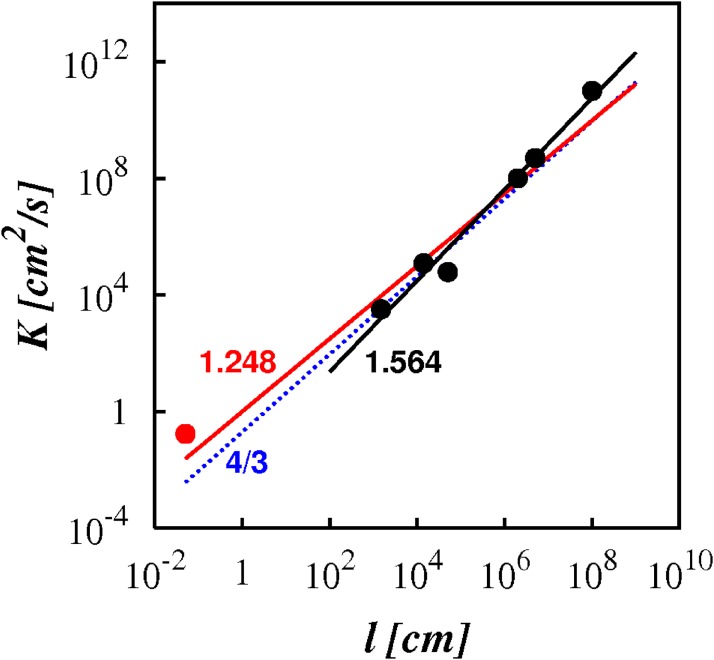
The turbulent pair diffusivity against separation, as log(*K*) against log(*l*). The symbols are the observational data reported by Richardson in 1926 (Table 1). The red filled circle (N1) is from of the molecular diffusion of oxygen into nitrogen. The black filled circles (N2-N7) are from geophysical settings. The dotted blue line is Richardson’s assumed locality scaling, $K∼\sigma_{l}^{4/3}$. The solid red line is the least square line of best fit to the entire 1926 dataset (N1-N7), $K∼\sigma_{l}^{1.248}$. The solid black line is the least square line of best fit to the revised dataset (N2-N7), $K∼\sigma_{l}^{1.564}$, and the coefficient of determination is, *R*^2^ = 0.97.

In 1926 Richrdson reported in the Proceedings of the Royal Society of London data on turbulent pair diffusivities collected from different sources [10], which is reproduced here with a brief description in Table 1. He plotted the turbulent diffusivity against the pair separation in log-log scale, shown as the red and black filled circles in Fig 1. Motivated by an attempt to unify pair diffusional processes across all possible scales in the limit of infinite Reynolds number, he made two important assumptions: firstly that the pair diffusion can be modeled by a diffusion-type equation, Eq (1), and secondly that the diffusion coefficient is scale dependent and can be modelled as a unique power law across all scales. From the collected data, he assumed the scaling *K* ∼ *l*^4/3^ as a reasonable fit (Fig 1, dotted blue line). This is not the least squares line of best fit to the data, it is just an approximation to the data. The actual line of best fit is, *K* ∼ *l*^1.248^ (Fig 1, solid red line).

**Table 1:** Datum number, source, diffussion coefficient *K*, and the length scale *l*.

Datum Number	Source	Diffusivity *K* [*cm*^2^/*s*]	Scale *l* [*cm*]
*N*1	Molecular diffusion of oxygen in to ntirogen [61]	1.7 × 10^−1^	5 × 10^−2^
*N*2	Anemometers 9 m above the ground [62]	3.2 × 10^3^	1.5 × 10^3^
*N*3	Anemometers 21-305 m above the ground [63]	1.2 × 10^5^	1.4 × 10^4^
*N*4	Pilot balloons 100-800 m above the ground [64, 65]	6 × 10^4^	5 × 10^4^
*N*5	Tracks of balloons in the atmosphere [66, 67]	1 × 10^8^	2 × 10^6^
*N*6	Volcano ash [66, 67]	5 × 10^8^	5 × 10^6^
*N*7	Diffusion from cyclones [68]	1 × 10^11^	1 × 10^8^

Later, Richardson and Stommel [58, 59] commenting on diffusion of floats in the sea noted that their new measurements were roughly consistent with the 1926 data. At the end of their paper they wrote, *‘Note added in proof.—After this manuscript was submitted the writers have read two unpublished manuscripts by C. L. von Weisaecker and W. Heisenberg in which the problem of turbulence for large Reynolds number is treated deductively with the result that they arrive at the 4/3-law. The agreement between von Weisaecker and Heisenberg’s deduction and our quite independent induction is a confirmation of both’*, see [60].

But they also observed that, *‘any power between ∼ l^1.3^ and ∼ l^1.5^ would be a tolerable fit to the data’*. Indeed, the 1926 data are from very different sources varying in length and time scales by orders of magnitude. Apart from this there are other physical processes whose impact on pair diffusion cannot be readily assessed, such as the effects of bouyancy, and the quasi two-dimensionality of the atmosphere. Similar concerns can be made regarding the 1948 and 1949 data. No attempt was made to assess the magnitude of these effects, so it is not surprising that such a wide error band in the power law scaling was given.

There is a more fundamental problem with the data. Data-point number N1 in Table 1 (Fig 1, red filled circle) is not from turbulence measurements at all, rather it is from studies of the molecular diffusion of oxygen into nitrogen and whose length scale is stated to be of the order of 10^−2^ cm. At such a small scale it cannot be turbulent, and certainly cannot contain an inertial subrange. This data-point is therefore disregarded as an outlier in the current investigation which strictly demands the existence of turbulence with an inertial subrange.

The remaining six data-points (Fig 1, black filled circles) are sound, coming from geophysical turbulence settings and certainly containing extended inertial subranges. The line of best fit to this new improved dataset (N2 to N7) displays an unequivocal non-local scaling, *K* ∼ *l*^1.564^ (Fig 1, solid black line). The coefficient of determination, *R*^2^ = 0.97, thus all the data-points lie close to line of best fit, Fig 1. The correct scaling must be close this; but even taking a reasonably wide margin of error, say between ∼ *l*^1.5^ and ∼ *l*^1.6^, this is outside the range noted by Richardson & Stommel.

The 1926 dataset is used as a guide to inspire a new idea—locality in the case of Richardson. Richardson and Stommel were aware of the wide margin of error in the data, but this did not prevent them from making a reasoned guess on the scaling power based on this data. The situation is similar here, except that the dataset has been corrected and now shows a clear shift towards non-locality, indicating that non-local diffusional processes cannot be ignored *a priori* in a general theory of turbulent pair diffusion, which is where we turn to in the next section.

## 4 A new theory

Like Richardson the focus here is on the diffusion coefficient *K*. Although the mean square separation 〈*l*^2^〉 is often the focus of diffusion studies, it is related directly to the diffussion coefficent by the exact relation, *K* = 0.5*d*〈*l*^2^〉/*dt*. If a general scaling, $K∼\sigma_{l}^{\gamma }$, is assumed then it yields 〈*l*^2^〉 ∼ *t*^*χ*^. For steady-state equilibrium turbulence *χ* is given by the relation,

$$\begin{array}{c} \chi =\frac{2}{2-\gamma } \end{array}$$ (7)

As such 〈*l*^2^〉 does not provide any additional information. We will therefore focus our attention mainly on *K* in the ensuing work, and refer to 〈*l*^2^〉 only where necessary. Our interest is scaling laws inside the inertial subrange, so we will ignore discussion of the viscous diffusion range and the long time Taylor diffusion. Furthermore, as our focus is on scalings rather than exact quantities we will supress unimportant constants wherever possible.

We will consider turbulence with generalized energy spectra of the form, *E*(*k*) ∼ *k*^−*p*^. The term ‘wavenumber’ will be used to denote both the vector, **k**, as well as its magnitude, *k* = |**k**|, and the context will make it clear. Generalised spectra of this type are routinely used in pair diffusion studies; it helps in understanding the balance of diffusional processes as the turbulence spectra changes. In the current work, generalised spectra will play an important role in developing the new theory, and also for testing the predictions of the theory [27]. The pair diffusion coefficient *K*(*l*, *p*) is now also a function of *p*. Eq (7) is true for generalised spectra as well, with *γ* = *γ*(*p*), and *χ* = *χ*(*p*).

### 4.1 The statement of the problem

The problem is to determine the pair diffusivity, *K* = 〈 **l** · **v** 〉, of an ensemble of pairs of fluid particles in a field of homogeneous turbulence with an energy density spectrum, *E*(*k*) containing an generalised inertial subrange, *E*(*k*) ∼ *k*^−*p*^, *k*_1_ ≤ *k* ≤ *k*_*η*_, for 1 < *p* ≤ 3, and such that *E*(*k*) → 0 as *k* → 0. The particles in a pair are located at **x**_1_(*t*) and **x**_2_(*t*) at time *t*, the pair displacement vector is **l**(*t*) = **x**_2_(*t*) − **x**_1_(*t*), and the pair separation is $l\left(t\right)=\sqrt{l_{1}^{2}+l_{2}^{2}+l_{3}^{2}}=\left|x_{2}\left(t\right)-x_{1}\left(t\right)\right|$. The initial separation at some earlier time, *t*_0_, is denoted by *l*_0_ = |**x**_2_(*t*_0_) − **x**_1_(*t*_0_)|. The turbulent velocity field is, **u**(**x**, t), and the particle velocities at time t are, respectively, **u**_1_(*t*) = **u**(**x**_1_(*t*), *t*) and **u**_2_(*t*) = **u**(**x**_2_(*t*), *t*), and the pair relative velocity is **v**(*t*) = **u**_2_(*t*) − **u**_1_(*t*).

We assume point source release, which in practical terms means that the initial pair separation must be close to the Kolmogorov length scale, *l*_0_ ≈ *η*. The particles will diffuse apart and eventually decorrelate with the initial conditions—they will ‘forget’ their initial conditions, (*l*_0_, *v*_0_), as Batchelor put it—after some travel time, $t_{l_{0}}$, when the pair is inside the inertial subrange. During this travel time, the pair will display ballistic motion with essentially constant velocity equal to the initial relative velocity *v* = *v*_0_; $\langle l^{2}\rangle =l_{0}^{2}+v_{0}^{2}{\left(t-t_{0}\right)}^{2}$. The transition from the ballistic regime to the explosive inertial subrange regime occurs on a time scale of the order of the eddy turnover time scale, $t_{l_{0}}\approx \tau_{0}\left(l_{0}\right)∼\varepsilon^{1/3}l_{0}^{1/3}$. If *l*_0_ ≈ *η* then the travel time is approximately equal to the Kolmogorov time microscale, $t_{l_{0}}\approx \varepsilon^{1/3}\eta^{1/3}$, which is very short. At much longer times we can ignore the ballistic regime because $t\gg t_{l_{0}}$ and 〈*l*^2^〉 ≫ *l*_0_.

Without loss of generality, it will also be assumed that, *t*_0_ = 0.

### 4.2 The mathematical framework

The main task here is to uncover what scales of turbulent motions contribute to the pair separation process when the pair separation *l*(*t*) is inside the inertial subrange. For this purpose, we decompose the pair diffusion coefficient in to its component scales using Fourier transforms. This is be obtained as follows.

The pair relative velocity is, **v**(**x**) = *d* **l** / *dt*, and *K* is defined as the ensemble average of the scalar product of **v** with **l**,

$$\begin{array}{c} K=\left\langle l\cdot v\right\rangle \end{array}$$ (8)

For homogeneous, isotropic, incompressible, reflectional and statistically stationary turbulence, the Fourier expression for the velocity field **u** is [69],

$$\begin{array}{c} u(x)=\int A(k)\;\text{exp}\;\left(ik\cdot x\right)\;d^{3}k \end{array}$$ (9)

where **A**(**k**) is the Fourier transform of the flow field, **k** is the associated wavenumber. The relative velocity **v** across a finite displacement **l**, is

$$\begin{array}{c} v(l)=u\left(x_{2}\right)-u\left(x_{1}\right). \end{array}$$ (10)

Using Eq (9) this is gives,

$$\begin{array}{c} v(l)=\int A(k)\left[\;\text{exp}\;(ik\cdot l)-1\right]\;\text{exp}\;(ik\cdot x_{1})\;d^{3}k. \end{array}$$ (11)

Taking the scalar product of **v** with **l**, and then the ensemble average 〈⋅〉 over particle pairs yields an expression for 〈**l** ·**v**〉. But the left hand side is a Lagrangian quantity, while the right hand side is an Eulerian quantity. We assume that the Lagrangian ensemble scales like the Eulerian ensemble; such a closure is often made in diffusion studies. Thomson & Devenish [70] for example make this assumption implicitly in their analysis of Lagrangian diffusion models.

We thus obtain a scaling for the pair diffusivity,

$$\begin{array}{c} K(l)∼\left\langle l\cdot v\right\rangle ∼\int \left\langle \left(l\cdot A\right)\left[\text{exp}(ik\cdot l)-1\right]\;\text{exp}\;(ik\cdot x_{1})\right\rangle d^{3}k. \end{array}$$ (12)

Because of homogeneity, the ensemble average removes the factor exp (*i* **k** · **x_1_**) without altering the scaling upon *l*. This gives,

$$\begin{array}{c} K(l)∼\int \left\langle \left(l\cdot A\right)\left[\;\text{exp}\;(ik\cdot l)-1\right]\right\rangle d^{3}k. \end{array}$$ (13)

Let *k_l_* = 1/*l* be the pair separation wavenumber; we follow the usual convention and replace *l* with the scaling, *l* ∼ *σ*_*l*_, throughout this work, so that

$$\begin{array}{c} k_{l}\left(t\right)∼\frac{1}{\sigma_{l}\left(t\right)}, \end{array}$$ (14)

where $\sigma_{l}^{2}=\langle l^{2}\rangle$. Note that *k_l_*(*t*) changes with time.

### 4.3 Turbulent transport processes

There are several transport processes that must to be considered. The largest eddies carry the smaller scales of motion, as depicted in Richardson’s poem ‘Big whorls have little whorls’. This is known as the sweeping effect, which means that in a frame of reference moving with these large scale convective motions the small scale transport process should proceed as if there were no large scale sweeping motions at all in a statistical sense. Thus pair diffusion inside the inertial subrange, which is a small scale process, should be the same in both frames of reference. This allows us to simplify the problem statement by working in the swept frame of reference by eliminating the large scale part of the energy spectrum by setting *E*(*k*) = 0 for *k* < *k*_1_. In this frame of reference, *E*(*k*) ∼ *k*^−*p*^, *k*_1_ ≤ *k* ≤ *k*_*η*_, and *E*(*k*) = 0 outside of this range of wavenumbers.

In the swept frame of reference, consider a particle pair whose separation distance is *l*(*t*), and the associated wavenumber is *k_l_* in Eq (14). In principle, all scales of motion in the inertial subrange could affect the pair diffusion process.

The eddies at wavenumbers that are much bigger than *k_l_* (the smallest scales of motion) probably act like molecular diffusion motion since the time scales are very small and the motions are somewhat randomised due to the high frequencies at these scales.

The eddies at wavenumbers close to *k_l_* are considered to be in the local wavenumber range to *k_l_*.

The large scale motions at wavenumbers that are much smaller than *k_l_* will be less correlated with the motions at scale *k_l_*. The largest of these scales become the sweeping scales which are statistically decoupled from the local wavenumbers other than carrying them en block. As the wavenumber spectrum is continuous, it follows that between the convecting scales and the wavenumbers local to *k_l_*, there could be a range of wavenumbers that are weakly correlated with the motions near *k_l_* while still being inside the inertial subrange, and this we call the non-local wavenumber range to *k_l_*.

In this picture, the hypothesis of locality corresponds to the assumption that the non-local scales of motion do not contribute to the inertial pair diffusion process.

Here, we do *not* make such an assumption.

The physical assumption adopted here about the nature of the diffusional processes that are occurring in the system is that there exist three broadly independent diffusional processes within the inertial subrange that potentially contribute to the pair diffusion process as a whole, each process acting from its own range of wavenumbers relative to the inverse pair separation wavenumber *k_l_*.

The three physical processes operate, respectively, from (i) the wavenumbers that are larger than *k_l_*, (ii) the wavenumbers that are local to *k_l_*, and (iii) the wavenumbers that are non-local to *k_l_*. The associated frequencies are, $\omega \left(k\right)\propto \sqrt{k^{3}E\left(k\right)}$, according to the usual assumption that the frequencies scale with the inverse turnover time of the eddy at wavenumber *k*. The local eddy frequency is, $\omega_{l}\propto \sqrt{k_{l}^{3}E\left(k_{l}\right)}$, and the local eddy turnover time is, *T_l_* ∼ 1/*ω*_*l*_.

The integral in Eq (13) is then the sum of three integrals over different wavenumber ranges which are defined as follows:

**s**: the small scales such that *k* ≫ *k_l_*, and |**k** · **l**| ≫ 1, and the associated frequencies are much larger than *ω*_*l*_, *ω*(*k*) ≫ *ω*_*l*_.
**l**: the local scales such that *k* ≈ *k_l_*, and |**k** · **l**| ≈ 1, and the associated frequencies are of the same order as *ω*_*l*_, *ω*(*k*) ≈ *ω*_*l*_.
**nl**: the non-local scales such that *k* ≪ *k_l_*, and |**k** · **l**| ≪ 1, and the associated frequencies are much smaller than *ω*_*l*_, *ω*(*k*) ≪ *ω*_*l*_.

Eq (13) then becomes,

$$\begin{array}{c} K(l)∼(\int_{nl}+\int_{l}+\int_{s})\left\langle \left(l\cdot A\right)\left(\;\text{exp}\;(ik\cdot l)-1\right)\right\rangle \;d^{3}k \end{array}$$ (15)

which we rephrase as,

$$\begin{array}{c} K(l)∼K^{nl}+K^{l}+K^{s}. \end{array}$$ (16)

We might approximate the integrand in Eq (15) by expanding the exponential term such that, exp (*i* **k** · **l**) − 1 ≈ *i* **k** · **l**. However, such an expansion is accurate only for low wavenumbers *k* ≪ *k_l_* where, |**k** · **l**| ⪡ 1. For local wavenumbers *k* ≈ *k_l_* where |**k** · **l**| ≈ 1, this expansion is only approximately true. For high wavenumbers *k* ≫ *k_l_* where |**k** · **l**| ≫ 1, this expansion is not accurate.

### 4.4 The physics of the small scales of motion

A simplification can be made with respect to the small scales of motion whose contribution to the diffusion process is,

$$\begin{array}{c} K^{s}\left(l\right)∼\int_{s}\left\langle \left(l\cdot A\right)\left(\;\text{exp}\;(ik\cdot l)-1\right)\right\rangle \;d^{3}k \end{array}$$ (17)

There is no need to evaluate this integral directly because the net ensemble effect can be assessed on physical grounds alone. *K*^*s*^ is an integral over high wavenumbers and represents the contribution from scales of turbulent motion which are much smaller than the pair separation, i.e., from *k* ≫ *k_l_*. The energy contained in these scales is very small if the energy spectrum decreases as k increases, such as an inverse power law of the type *E*(*k*) ∼ *k*^−*p*^, with *p* > 1.

Furthermore, these small scales are associated with the unsteadiness of high frequencies, *ω* ≫ *ω*_*l*_. Statistically, these high frequency motions induce random and rapid changes in the direction and the magnitude of the pair displacement vector.

Overall, the net statistical impact of these high frequency, low energy, and random turbulent velocity fluctuations on the pair diffusion process is expected to be extremely small, i.e., *K*^*s*^ ≪ *Max*(*K*^*l*^, *K*^*nl*^). Thus, we assume that *K*^*s*^ ≈ 0 and *K*^*s*^ will be ignored from now on.

### 4.5 The physics of the local and non-local scales of motion

With the effect of the small scale contributions eliminated, the simplified expression for the pair diffusivity is,

$$\begin{array}{ll} K\left(l\right) & ∼K^{nl}\left(l\right)+K^{l}\left(l\right)\\ & ∼\underset{nl}{\int }\langle \left(l\cdot A\right)\left(\text{exp}\left(ik\cdot l\right)-1\right)\rangle \;d^{3}k+\underset{l}{\int }\langle \left(l\cdot A\right)\left(\text{exp}\left(ik\cdot l\right)-1\right)\rangle \;d^{3}k \end{array}$$ (18)

The expansion of the exponential in the integrand to leading order is accurate only in the non-local range because |**k** · **l**| ⪡ 1. In the local range where |**k** · **l**| ≈ 1, such an expansion is only approximately true because the local eddies are moderately unsteady with frequencies that are of the same order of magnitude as, *ω*_*l*_. The effect on the local diffusion process is assumed to be likewise moderate.

We will assume that the ensemble effect of the unsteadiness from the local wavenumbers is to reduce the magnitude of *K*^*l*^, but without altering its overall scaling behaviour. Then, the expansion, exp (*i* **k** · **l**) − 1 ≈ *i* **k** · **l**, can be used in Eq (18) but with the magnitude of *K*^*l*^ reduced by some factor, *F_l_* ≲ 1—this constant is smaller than unity, but not too small. Then (18) becomes,

$$\begin{array}{c} K(l)∼\int_{nl}\left\langle \left(l\cdot A\right)\left(ik\cdot l\right)\right\rangle d^{3}k+F_{l}\int_{l}\left\langle \left(l\cdot A\right)\left(ik\cdot l\right)\right\rangle d^{3}k. \end{array}$$ (19)

*F_l_* = *F_l_*(*p*, *R_l_*, *C*) is not expected to be a universal constant because it will depend upon various parameters, like *p*, and *R_l_* = *k_l_*/*k*_1_ (which is the size of the inertial subrange relative to the particle pair separation), and also implicitly upon the size of an eddy in wavenumber space, *C* (to be defined later). After absorbing constants, the integrands in Eq (19) become,

$$\begin{array}{c} \left\langle l^{2}|A||k|\cos\left(\alpha \right)\cos\left(\beta \right)\right\rangle \end{array}$$ (20)

where *α* is the angle between **l** and **A**, and *β* is the angle between **l** and **k**. For isotropic random fields averaging (20) over all directions, again, does not affect the scaling behaviour. *α* and *β* are not uniformly distributed in all direction, it is well known that **l** aligns preferentially in the positive strain directions, and this ensures that the ensemble average above is non-zero.

Retaining the angled brackets 〈⋅〉 to include averaging over all directions, Eq (19) with Eq (20) simplifies to,

$$\begin{array}{c} K(l)∼\int \int_{nl}\left\langle l^{2}ak\right\rangle dkdA\left(k\right)+F_{l}\int \int_{l}\left\langle l^{2}ak\right\rangle dkdA\left(k\right) \end{array}$$ (21)

where *a* = |**A**|, and *dA*(*k*) is the element of surface area at radius *k* in wavenumber space.

If the closure, 〈*l*^2^*ak*〉 ∼ 〈*l*^2^〉〈*ak*〉, is assumed, then upon integrating over the surface area this integral becomes

$$\begin{array}{c} K(l)∼(\int_{nl}\left\langle ak\right\rangle dk+F_{l}\int_{nl}\left\langle ak\right\rangle dk)\left\langle l^{2}\right\rangle . \end{array}$$ (22)

Note that 〈*ak*〉 ≠ 0 even though the vectors **k** and **A** are orthogonal because *a* and *k* are magnitudes.

∫〈*a*^2^〉*dA*(*k*) is the energy density per unit wavenumber averaged over all directions [69] and scales like ∼ *E*(*k*)/*k*. If the closure, $\int \langle ak\rangle dA\left(k\right)∼k\sqrt{\int \langle a^{2}\rangle dA\left(k\right)}$ is assumed then Eq (22) becomes,

$$\begin{array}{c} K(l)∼\sigma_{l}^{2}\int_{nl}\sqrt{kE(k)}dk+\sigma_{l}^{2}F_{l}\int_{nl}\sqrt{kE(k)}dk \end{array}$$ (23)

As a further check, this can also be derived as follows. The velocity variance from the scales *k* to *k* + *dk* is *E*(*k*)*dk*, and the variance of velocity gradient is *k*^2^ *E*(*k*)*dk*. The particle pair velocity variance is, ∼ 〈*l*^2^〉*k*^2^ *E*(*k*)*dk*. The time scale of eddies of wavenumber *k* is $1/\sqrt{k^{3}E\left(k\right)}$. So the incremental contribution to the diffusivity from these scales is the pair velocity variance times the time scale, $dK∼\langle l^{2}\rangle \sqrt{kE(k)}dk$, which leads to Eq (23).

To make further progress the actual form of the turbulence spectrum must be specified. In this work, the focus is on turbulence which contains an inertial subrange. For pair diffusion statistics, as mentioned earlier, this is implemented by working in the swept frame of reference by setting the spectrum in the large energy scales to zero, *E*(*k*) = 0 for *k* < *k*_1_, and assuming an inverse power-law energy spectrum in the inertial subrange,

$$\begin{array}{c} E(k)=C_{k}\varepsilon^{2/3}L^{5/3-p}k^{-p},\;k_{1}<k<k_{\eta },\;1<p\leq 3 \end{array}$$ (24)

where *C_k_* is a constant. A large length scale *L* is necessary for dimensional consistency. *L* scales with some length scale that is characteristic of the large energy scales, such as the integral length scale, or the Taylor length scale.

With the spectrum in Eq (24), and with $\sigma_{l}^{2}=\langle l^{2}\rangle$, Eq (23) becomes,

$$\begin{array}{c} K(l,p)∼\varepsilon^{1/3}L^{(5/3-p)/2}(\int_{nl}k^{(1-p)/2}dk+F_{l}\int_{l}k^{(1-p)/2}dk)\sigma_{l}^{2} \end{array}$$ (25)

This is the most general expression for *K* that can be derived from the present analysis without any *a priori* assumption regarding locality.

### 4.6 Validation: The locality limit

To check the effectiveness of the mathematical approach adopted here in deriving Eq (25), the locality limit from this expression must first be validated against Richardson’s locality hypothesis for which there is a known theory.

The assumption of locality means that the non-local (first) term on the right hand side in Eq (25) is ignored. To evaluate the remaining local integral we assume a cut-off wavenumber $k_{l}^{*}$ that separates the local and non-local ranges, and such that $k_{1}⪡k_{l}^{*}<k_{l}$. Thus, $k_{1}\leq k<k_{l}^{*}$ defines the non-local kernel (range) to *k_l_* in wavenumber space, and $k_{l}^{*}\leq k\leq k_{l}$ defines the local kernel to *k_l_*.

Using the scaling $\langle l^{2}\rangle ∼1/k_{l}^{2}$ the local integral in (25) yields,

$$\begin{array}{c} K^{l}\left(l,p\right)∼\frac{2F_{l}\varepsilon^{1/3}}{3-p}L^{\left(5/3-p\right)/2}k_{l}^{\left(3-p\right)/2}\left(1-{\left(\frac{k_{*}}{k_{l}}\right)}^{\left(3-p\right)/2}\right)\frac{1}{k_{l}^{2}} \end{array}$$ (26)

Let the size of the locality kernel with respect to *k_l_* be defined by,

$$\begin{array}{c} C(p,R_{l})=\frac{k_{l}}{k_{l}^{*}}, \end{array}$$ (27)

where *C* is finite and greater than unity. The size of the inertial subrange with respect to *k_l_* is,

$$\begin{array}{c} R_{l}\left(t\right)=\frac{k_{l}\left(t\right)}{k_{1}}, \end{array}$$ (28)

Note that *R_l_*(*t*) changes with time because *k_l_*(*t*) changes with time. *R_l_*, is related to a local Reynolds number through, $Re_{l}∼R_{l}^{4/3}$. Thus, all dependencies on *R_l_* could be replaced by dependencies on *Re_l_*, e.g. *C* = *C*(*p*, *Re_l_*). We will use *R_l_* in the current analysis.

Then Eq (26) becomes,

$$\begin{array}{c} K^{l}\left(l,p\right)∼\frac{2F_{l}}{3-p}\varepsilon^{1/3}L^{\left(5/3-p\right)/2}k_{l}^{-\left(1+p\right)/2}\left(1-{\left(\frac{1}{C}\right)}^{\left(3-p\right)/2}\right). \end{array}$$ (29)

Absorbing constants, this simplifies to,

$$\begin{array}{c} K^{l}\left(l,p\right)∼F_{l}\varepsilon^{1/3}L^{(5/3-p)/2}\sigma_{l}^{\gamma^{l}\left(p\right)},\;\text{where}\;\gamma^{l}\left(p\right)=\left(1+p\right)/2,\;1<p\leq 3 \end{array}$$ (30)

Eq (30) reproduces the correct generalized locality scaling, *γ*^*l*^(*p*) = (1 + *p*)/2, [30, 71, 72]. For Kolmogorov turbulence, *p* = 5/3, this gives, $K∼\sigma_{l}^{4/3}$, which recovers the Richardson’s 4/3-scaling law.

### 4.7 The influence of non-local scales

*A priori* there is no reason to neglect the non-local term, which is the first term on the right hand side in Eq (25),

$$\begin{array}{c} K^{nl}\left(l,p\right)∼\varepsilon^{1/3}L^{(5/3-p)/2}(\int_{nl}k^{(1-p)/2}dk)\sigma_{l}^{2} \end{array}$$ (31)

This is equivalent to strained relative motion where each scale of turbulence in the non-local wavenumber range, $k_{1}<k<k_{l}^{*}$, will set up a straining field in the neighbourhood of *k_l_* which will alter the rate of increase of the pair separation. Previous theories have assumed that such non-local effects are negligible. However, there are three factors that suggest that this may be an oversimplification.

Firstly, the non-local wavenumbers, $k_{1}<k<k_{l}^{*}$, possess much greater energies than at the local separation wavenumber *k_l_*, and this will increase their relative influence in the pair diffusion process.

Secondly, the time scale of the non-local scales, *T*^*nl*^(*k*), are much larger than the local turnover time scale, *T*^*nl*^(*k*) ≫ *T_l_* ∼ 1/*ω*_*l*_, so the straining fields set up by non-local wavenumbers will persist for longer times than *T_l_*, which will enhance their effectiveness.

Thirdly, although an individual non-local wavenumber contribution is weak, the integral is over a large part of the energy spectrum. Again, this will enhance the effectiveness of the non-local scales in the pair diffusion process.

Taken together, there is a fair chance that the total non-local contributions could be significant at least in some parameter range.

Changing variables in the integrand in Eq (31) to *s* = *k*/*k*_1_, yields

$$\begin{array}{c} K^{nl}\left(l,p\right)∼\varepsilon^{1/3}L^{(5/3-p)/2}k_{1}^{(3-p)/2}(\int_{nl}s^{(1-p)/2}ds)\sigma_{l}^{2}. \end{array}$$ (32)

Inside the integral the non-local wavenumbers in range of integration $\left[k_{1},k_{l}^{*}\right]$ do not scale with *k_l_* so the integral is a definite integral producing a non-dimensional number, *S_nl_* = *S_nl_*(*p*, *R_l_*, *C*). *S_nl_* is not expected to be a universal constant. This gives,

$$\begin{array}{c} K^{nl}\left(l,p\right)∼S_{nl}\varepsilon^{1/3}L^{(5/3-p)/2}k_{1}^{(3-p)/2}\sigma_{l}^{2}. \end{array}$$ (33)

If the upper end of the inertial subrange is assumed to scale with the large scales, *k*_1_ ∼ 1/*L*, then this simplifies further to,

$$\begin{array}{c} K^{nl}\left(l,p\right)∼S_{nl}\varepsilon^{1/3}L^{-2/3}\sigma_{l}^{\gamma^{nl}\left(p\right)},\;\text{with}\;\gamma^{nl}\left(p\right)=2,\;1<p\leq 3 \end{array}$$ (34)

*γ*^*nl*^(*p*) is the non-locality scaling, and it is always equal to 2, independent of *p*. *K*^*nl*^ is thus always strain dominated being proportional to $\sigma_{l}^{2}$.

### 4.8 A general expression for the pair diffusivity

The overall expression for the turbulent pair diffusion coefficient is therefore,

$$\begin{array}{c} K(l,p)∼O(F_{l}\varepsilon^{1/3}L^{(5/3-p)/2}\sigma_{l}^{\gamma^{l}\left(p\right)})+O(S_{nl}\varepsilon^{1/3}L^{-2/3}\sigma_{l}^{2}),\;1<p\leq 3, \end{array}$$ (35)

or simply,

$$\begin{array}{c} K(l,p)∼O(\sigma_{l}^{\gamma^{l}\left(p\right)})+O(\sigma_{l}^{\gamma^{nl}\left(p\right)}),\;1<p\leq 3 \end{array}$$ (36)

where,

$$\begin{array}{c} \gamma^{l}\left(p\right)=(1+p)/2\;\;\text{local}\;\text{scaling} \end{array}$$ (37)

$$\begin{array}{c} \gamma^{nl}\left(p\right)=2\;\;\;\;\text{non-local}\;\text{scaling} \end{array}$$ (38)

Eq (36) with Eqs (37) and (38) means that turbulent pair diffusion is governed by a local diffusional process, and a non-local diffusional process which is produced by straining fields set up by the large scales.

But, what is the relative balance of their contributions? What does Eq (36) imply for the overall scaling for *K*?

## 5 The scaling laws for *K*

To address these questions, we need to first establish some general properties of the function *C* in Eq (27).

$C=k_{l}/k_{l}^{*}$ is a fundament physical quantity. It represents the range of wavenumbers close to *k_l_* over which motions are well correlated with motions at scale *k_l_*. It is a type of non-dimensionalised correlation scale for particle pairs. We call it the locality kernel, or we may call it the locality correlation length. In essence, this means that motions with scales within this kernel are deemed to scale locally with *k_l_*, but motions with scales outside this kernel do not scale with *k_l_*.

*C* is finite, 1 < *C* < ∞, and it depends on *R_l_*, and on the energy spectrum *E*(*k*) ∼ *k*^−*p*^.

*E*(*k*) becomes less steep as *p* → 1, so there is relatively more energy in the smaller scales when *p* approaches 1; this indicates that *C* must increase in this limit, *C* ≫ 1. On the other hand, as *p* → 3, then *C* → 1 because all scales of motion act non-locally so that $k_{l}^{*}\to k_{l}$.

The balance of the local and non-local diffusion is defined as the ratio, *M_K_* = *K*^*nl*^/*K*^*l*^. *M_K_* is a function of, *p*, *R_l_*, and *C*, and from Eqs (29) to (34) we obtain,

$$\begin{array}{c} M_{K}\left(p,R_{l},C\right)=\frac{K^{nl}}{K^{l}}∼\frac{\left(1-{\left(\frac{C}{R_{l}}\right)}^{\left(3-p\right)/2)}\right)}{F_{l}\left(C^{\left(3-p\right)/2)}-1\right)} \end{array}$$ (39)

Usually, we expect that *C* ≫ 1, which simplifies the above to,

$$\begin{array}{c} M_{K}\left(p,R_{l},C\right)∼\frac{1}{F_{l}}(\frac{1}{C^{\left(3-p)/2\right)}}-\frac{1}{R_{l}^{\left(3-p)/2\right)}}) \end{array}$$ (40)

Furthermore, in subranges that are bigger than the locality kernel, *R_l_*/*C* ≫ 1, this simplifies further to,

$$\begin{array}{c} M_{K}\left(p,R_{l},C\right)\to \frac{1}{F_{l}}\frac{1}{C^{\left(3-p)/2\right)}},\;\text{as}\;R_{l}/C\to \infty \end{array}$$ (41)

We can now obtain some properties of *M_k_*, and hence predict some scalings for the pair diffusion coefficient.

### 5.1 Scaling for Kolmogorov turbulence *p* = 5/3

Before we look at the general case, 1 < *p* ≤ 3, it is instructive to look first at the Kolmogorov turbulence case *p* = 5/3. Then in the limit of infinite subrange *R_l_*/*C* → ∞, Eq (41) is,

$$\begin{array}{c} M_{K}\left(R_{l},C\right)\to \frac{1}{F_{l}}\frac{1}{C^{2/3}}. \end{array}$$ (42)

This is a finite limit and we can therefore expect the balance between local and non-local processes to be finite. In other words, neither one process nor the other will be completely dominant.

But in the limit of short finite subranges such that *R_l_*/*C* → 1, i.e. *R_k_*/*C* > *R_l_*/*C* ≈ 1, Eq (40) gives,

$$\begin{array}{c} M_{K}\left(R_{l},C\right)\approx \frac{1}{F_{l}}(\frac{1}{C^{2/3}}-\frac{1}{R_{l}^{2/3}})\approx 0. \end{array}$$ (43)

Thus, for reasonably short inertial subranges, the non-local processes are negligible and we expect locality to be completely dominant, at least approximately.

**Fig 2:**
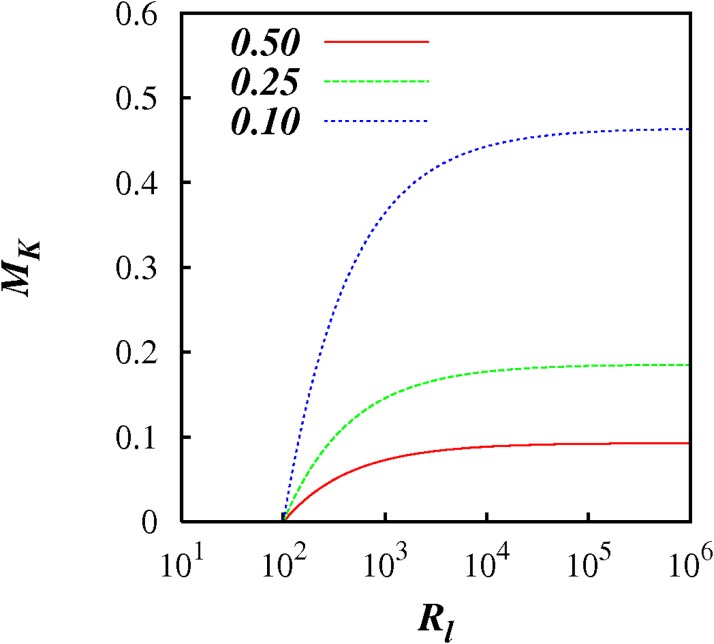
*M_K_* against log(*R_l_*), from Eq (40) with *p* = 5/3, *C* = 100, and *F_l_* = 0.1, 0.25, 0.5 as indicated.

For an ad hoc value of *F_l_* = 0.25, and *C* = 100, we obtain *M_k_* ≈ 0.15 in the limit of *R_l_*/*C* → ∞. This is illustrated in Fig 2, which shows plots of *M_k_* against log(*R_l_*), for *p* = 5/3, *C* = 100, and for three choices of *F_l_* = 0.1, 0.25, 0.5. These are only estimates, but it illustrates the finite balance between the local and non-local diffusional processes.

If the inertial subrange is too short, *R_l_* < *C*, then the above analysis breaks down and we do not observe any kind of inertial range scaling.

In summary, for asymptotically infinite inertial subrange (Reynolds number) we obtain a finite balance between local and non-local diffusional processes; but in the limit of short inertial subrange locality is restored because the non-local processes are negligible.

Do these limiting cases also exist for general spectra?. We investigate this in the next section.

### 5.2 Infinite inertial subrange *R_k_* → ∞ (*Re* → ∞)

First, we derive the scaling laws for the pair diffusion coefficient in the limit of infinite inertial subrange (infinite Reynolds number). Henceforth we assume that, *F_l_* < 1, and *R_l_* > *C* > 1, and *R_k_* > *R_l_*, unless otherwise stated.

From Eq (40), as *p* → 1, then *M_K_* → (1/*C* − 1/*R_l_*)/*F_l_*. For large inertial subranges, *R_k_*/*C* > *R_l_*/*C* ≫ 1, we obtain *M_K_* → 1/*F_l_* *C* ≪ 1, and therefore locality dominates in this limit as expected.

From Eq (39), it is easily shown that as *p* → 3, then *M_K_* → ∞, and therefore non-locality always dominates in this limit, again as expected. (Exact non-locality corresponds to *C* = 1).

In the intermediate range, 1 < *p* < 3, although the balance *M_K_* cannot be obtained quantitatively without the explicit form of *F_l_* and *C* as a functions of *p* and *R_l_*, it is instructive to investigate *M_K_* with some test functions for *C* to uncover some general trends in *M_K_*.

For this purpose, we simplify further by assuming an ad hoc value of *F_l_* = 0.25. We choose smooth exponential type test functions, *C*(*p*) = 1 + 100(exp(−*A*(*p* − 1)) − exp(−2*A*)), such that *C* ≫ 1 at *p* = 1, and *C* = 1 at *p* = 3. *A* > 0 is a given constant. *M_K_* was calculated using *C*(*p*) in (39).

**Fig 3:**
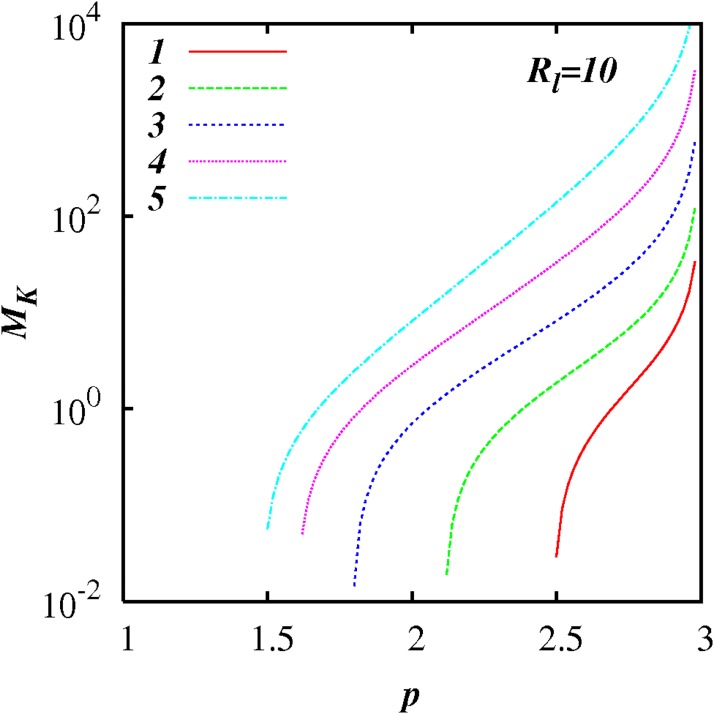
log(*M_K_*) against *p*, Eq (39), with exponential test functions, *C*(*p*) = 1 + 100(exp(−*A*(*p* − 1) − exp(−2*A*)), and *F_l_* = 0.25, and *R_l_* = 10^1^. Five cases are shown, *A* = 1, 2, 3, 4, 5.

**Fig 4:**
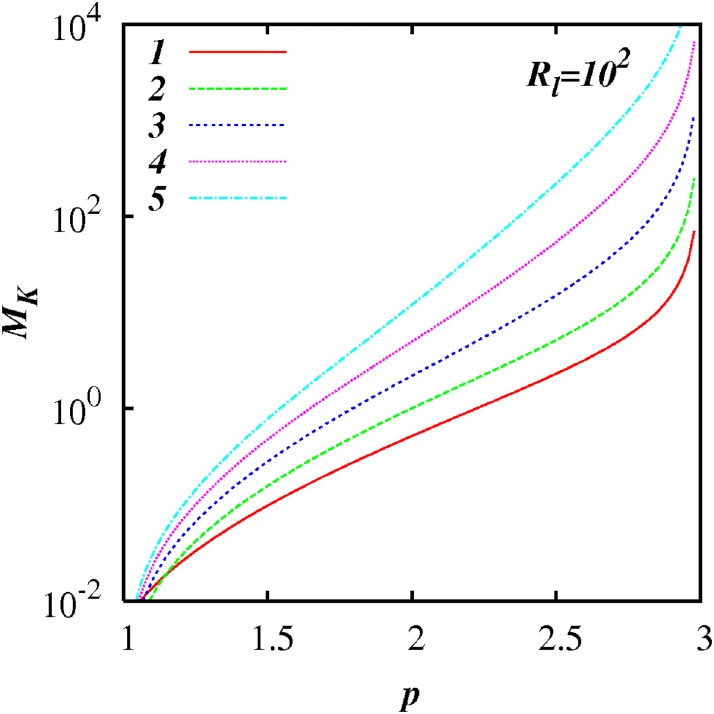
Similar to Fig 3, except for *R_l_* = 10^2^.

**Fig 5:**
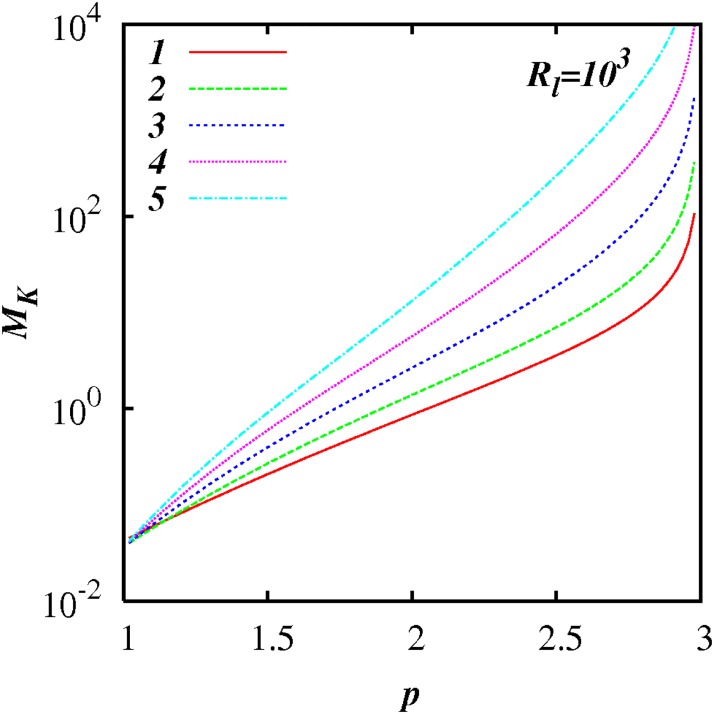
Similar to Fig 3, except for *R_l_* = 10^3^.

**Fig 6:**
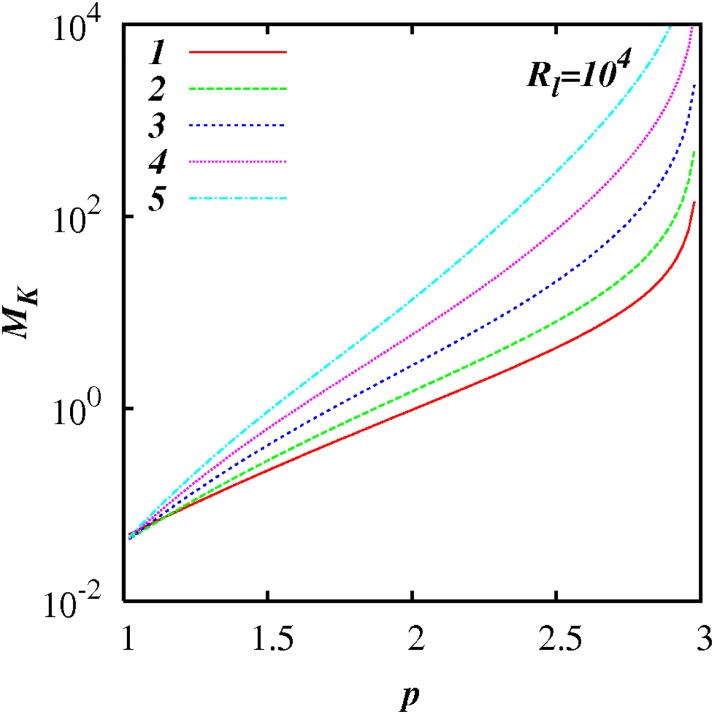
Similar to Fig 3, except for *R_l_* = 10^4^.

Figs 3 to 6 show plots of log(*M_k_*) against *p* for different inertial subrange size, respectively, *R_l_* = 10^1^, 10^2^, 10^3^, and 10^4^, for five selected values of *A* = 1, 2, 3, 4, 5 as shown. In all the cases, *M_K_* ≪ 1 as *p* → 1, and *M_K_* ≫ 1 as *p* → 3; and *M_K_*(*p*) increases smoothly and monotonically as *p* increases from 1 to 3.

*M_k_* approaches relatively close to 1 in a range of *p* close to *p* = 5/3—see the case for *A* = 2 in Fig 5 for example.

**Fig 7:**
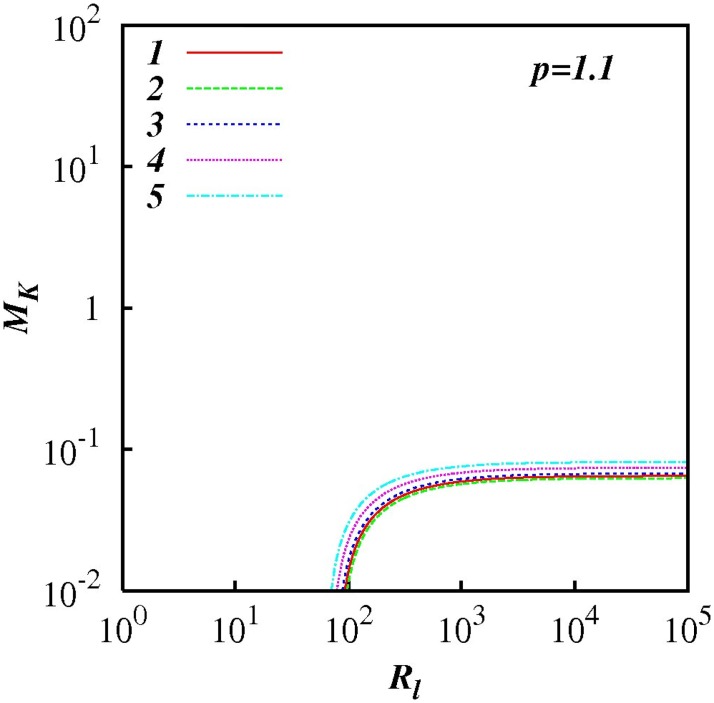
log(*M_K_*) against *p*, Eq (39), with exponential test functions, *C*(*p*) = 1 + 100(exp(−*A*(*p* − 1) − exp(−2*A*)). *F_l_* = 0.25 and *p* = 1.1. Five cases are shown, *A* = 1, 2, 3, 4, 5.

**Fig 8:**
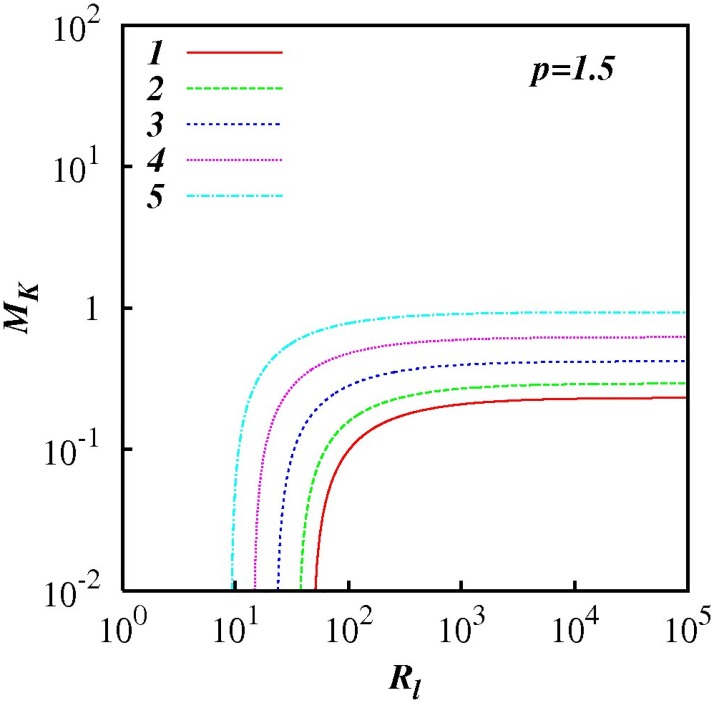
Similar to Fig 7, except for *p* = 1.5.

**Fig 9:**
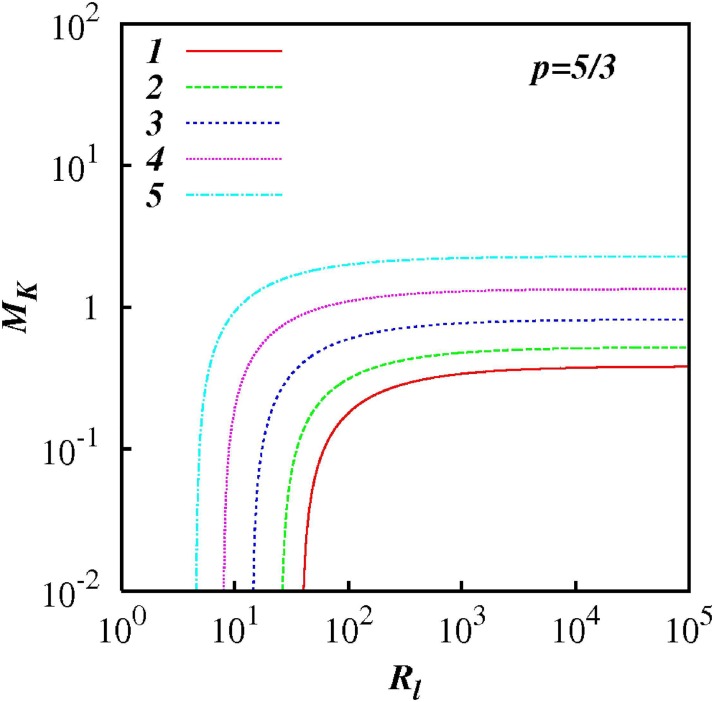
Similar to Fig 7, except for *p* = 5/3.

**Fig 10:**
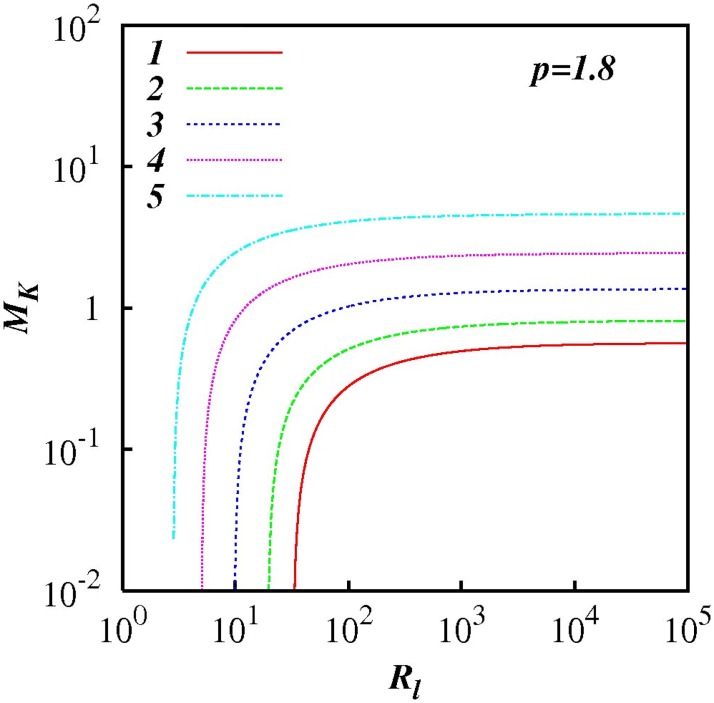
Similar to Fig 7, except for *p* = 1.8.

**Fig 11:**
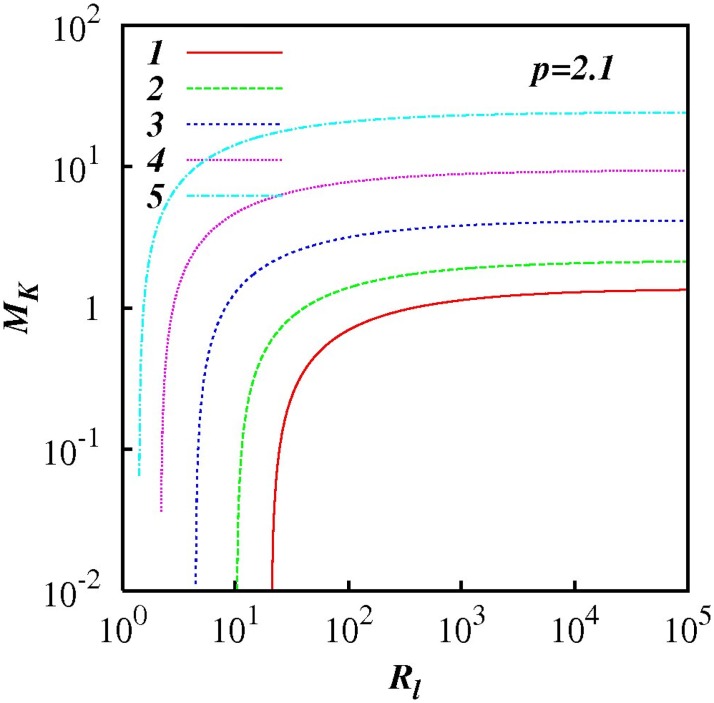
Similar to Fig 7, except for *p* = 2.1.

**Fig 12:**
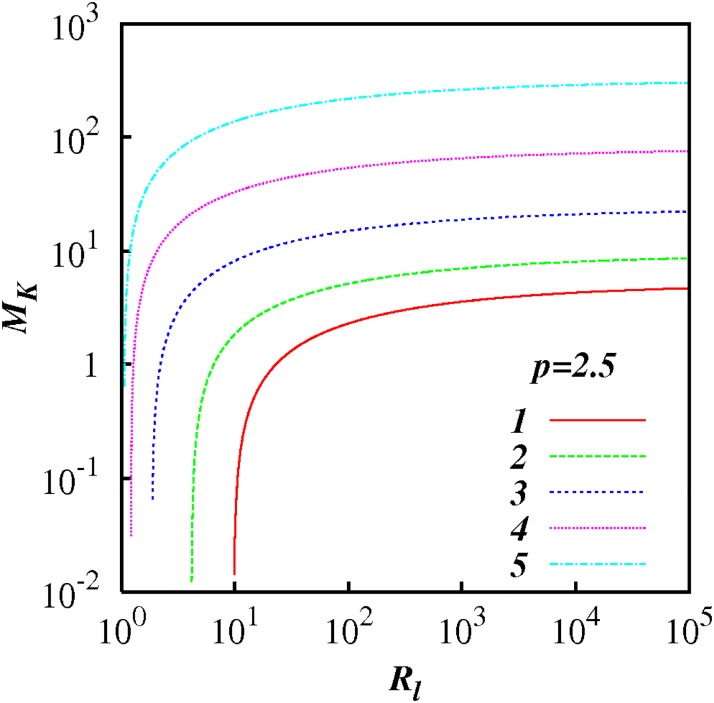
Similar to Fig 7, except for *p* = 2.5.

For any *p* in the range 1 < *p* < 3, *M_k_* increases as *R_l_* increases indicting the increasing influence of non-local processes. This feature is highlighted in Figs 7 to 12 which show plots of log(*M_k_*) and against log(*R_l_*) for selected spectra, respectively *p* = 1.1, 1.5, 5/3, 1.8, 2.1 and 2.5. For the Kolmogorov spectrum *p* = 5/3, in Fig 8, we observe that *M_k_* is close to unity for *R_l_* > 10^3^ indicating that both local and non-local diffusional processes could play significant roles in large inertial subranges.

We can draw the following conclusions for large inertial subranges where *R_k_*/*C* > *R_l_*/*C* ≫ 1.

Firstly, as *p* → 1 then *M_K_* ≪ 1, and therefore *K*^*nl*^ ≪ *K*^*l*^, yielding the locality limit,

$$\begin{array}{c} K(l,p)\to K^{l}\left(l,1\right)∼\sigma_{l}^{1}\;\text{as}\;p\to 1. \end{array}$$ (44)

Secondly, as *p* → 3 then *M_K_* ≫ 1, and therefore *K*^*nl*^ ≫ *K*^*l*^, yielding the non-locality limit,

$$\begin{array}{c} K(l,p)\to K^{nl}\left(l,3\right)∼\sigma_{l}^{2}\;\text{as}\;p\to 3. \end{array}$$ (45)

Thirdly, *M_K_*(*l*, *p*) is a smooth function of *p* in the range 1 < *p* ≤ 3 and it increases smoothly and monotonically as *p* increases smoothly from 1 to 3; this indicates that the non-local scales exert increasingly stronger influence until they are completely dominant at *p* = 3.

Fourthly, The influence of the non-local process increases at any *p* in the rangel 1 < *p* < 3 as the size of the inertial subrange increases.

From these considerations, there is a fair chance that in the critical range of spectra close to Kolmogorov *p* = 5/3 both the local and non-local processes could exert significant influence in the diffusional process.

We now apply an extension of Richardson’s second hypothesis, that at any *p* the diffusion coefficient is described by a single power, say *γ*(*p*), across all scales in the limit of infinite inertial subrange. This is plausible because self-similarity is exact in this limit—that is you cannot tell the difference in the scaling at different scales, so you must observe the same power at all scales.

Since *M_K_* is a smooth and continuous function of *p* in the range 1 < *p* ≤ 3, then *K*(*l*, *p*) must also be a smooth and continuous function of *p* in this range and it must display a smooth transition between its asymptotic limits, $K\left(l,1\right)∼\sigma_{l}^{1}$ and $K\left(l,3\right)∼\sigma_{l}^{2}$, as *p* passes smoothly from 1 to 3.

Is it possible that either one of the local or non-local processes dominates throughout the inertial subrange for any given *p*? That would imply a discontinuous jump between locality and non-locality scalings at some value of *p* in order to satisfy the asymptotic limiting cases in (44) and (45), but this would violate the continuity in *K* as a function of *p*.

Thus, *K*(*l*, *p*) must be a power law scaling which is intermediate between the purely local and non-local scalings,

$$\begin{array}{c} K(l,p)∼\sigma_{l}^{\gamma (p)},\;\text{as}\;R_{k}/C\to \infty \;\text{with}\;\gamma^{l}\left(p\right)<\gamma \left(p\right)<\gamma^{nl}\left(p\right),\;1<p<3. \end{array}$$ (46)

The scaling powers *γ*(*p*) are such that, as *p* → 1 then *γ*(*p*) → 1, and as *p* → 3 then *γ*(*p*) → 2. Globally, 1 < *γ*(*p*) ≤ 2. Furthermore, *γ*(*p*) must transform smoothly between these limiting cases as *p* goes from 1 to 3.

Eq (46) is the main prediction of the new theory in the limit *R_k_*/*C* → ∞, with *γ*^*l*^(*p*) and *γ*^*nl*^(*p*) given by (37) and (38). This is equivalent to the mean square separation scaling, 〈*l*^2^〉 ∼ *t*^*χ*(*p*)^, with *χ*(*p*) given by Eq (7). Eq (7) is a non-linear relation between *γ* and *χ*, so small changes in *γ* could produce large changes in *χ*.

For Kolmogorov turbulence, *E* ∼ *k*^−5/3^, the new theory predicts that, *γ* > 4/3, and *χ* > 3.

For turbulence with intermittency *μ*_*I*_ > 0, such that *E* ∼ *k*^−(5/3+*μ*_*I*_)^, the scaling is again greater than from a purely local theory,

$$\begin{array}{cc} \gamma_{\mu_{I}}>\gamma_{\mu_{I}}^{l} & =4/3+\mu_{I}/2. \end{array}$$ (47)

It is interesting to note that even under the classical locality assumption, in real turbulence with intermittency *p* = 1.72 we should obtain the scaling *γ*^*l*^ = 1.36, and *χ*^*l*^ = 3.125; thus, the classical RO-*t*^3^ regime does not actually exist! This is important for another reason because it gives us a way of testing how close current DNS is to at the locality limit—see Discussion, Section 6.

A non-local 4/3-law, $K∼\sigma_{l}^{4/3}$, is precited for some spectrum, $E\sim k^{-p_{*}}$, where p_*_ < 5/3 and *γ*(p_*_) = 4/3. This is equivalent to 〈*l*^2^〉 ∼ *t*^3^, with *χ*(p_*_) = 3, which is a new non-Richardson-Obukhov *t*^3^-regime for the mean square separation.

We define *M*_*γ*_(*p*) to be the ratio of the scaling power *γ*(*p*) to and local scaling powers *γ*^*l*^(*p*),

$$\begin{array}{c} M_{\gamma }\left(p\right)=\frac{\gamma (p)}{\gamma^{l}\left(p\right)}. \end{array}$$ (48)

*M*_*γ*_(*p*) is equal to 1 at both *p* = 1 and *p* = 3, and since *M*_*γ*_ > 1 in the range 1 < *p* < 3, then there must be a maximum in *M*_*γ*_ at some *p* = *p_m_* for an energy spectrum *E* ∼ *k*^−*pm*^, where 1 < *p_m_* < 3.

Richardson’s 1926 dataset, Fig 1, from real geophysical turbulence (i.e. including intermittency) suggests a scaling of, $\gamma_{\mu_{I}}\approx 1.564$, i.e. $K_{\mu_{I}}∼\sigma_{l}^{1.564}$. The theory developed here in Eq (46) is more consistent with this data than previous theories. However, not all large-scale measurements agree with this, as discussed in Section 2.

**Fig 13:**
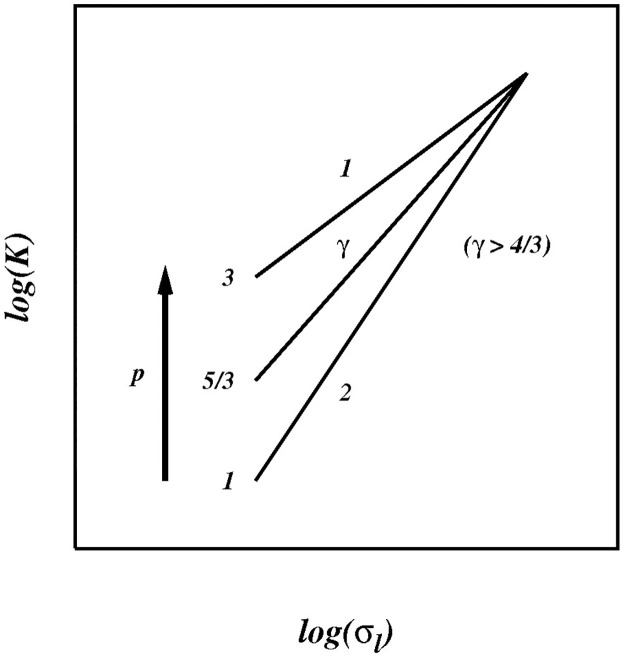
Sketch of the pair diffusion coefficient *K* against *σ*_*l*_ for 1 < *p* ≤ 3, as predicted from the current theory in the asymptotic limit of very large inertial range *R_k_*/*C* → ∞, (or *Re* → ∞), Eq (46).

The preditions for asymptotically infinite inertial subrange *R_k_* → ∞ is summarised in the sketch in Fig 13 which shows the predicted log-log plots of *K* against *σ*_*l*_ as *p* increases from 1 to 3.

Finally, we remark that corrections from the ends of the inertial subrange may modify some of the regimes predicted above. Ultra-violet corrections from the high wavenumber close to *k*_*η*_, and infra-red corrections from the low wavenumbers close to *k*_1_ may penetrate some way in to the inertial subrange, but the degree of penetration is unknown. Nevertheless, for very large subranges, we still expect to observe unambigous inertial range scalings over an extended inner part of the subrange.

### 5.3 Exposing the local process—Short inertial subrange, *R_k_* ≪ ∞ (*Re* ≪ ∞)

Eq (36) means that turbulent pair diffusion is governed by two broadly *independent* local and non-local processes. This excites a compelling question; are there circumstances in which we can ‘turn off’ either one of the processes thus exposing the other process explicitly?

**Fig 14:**
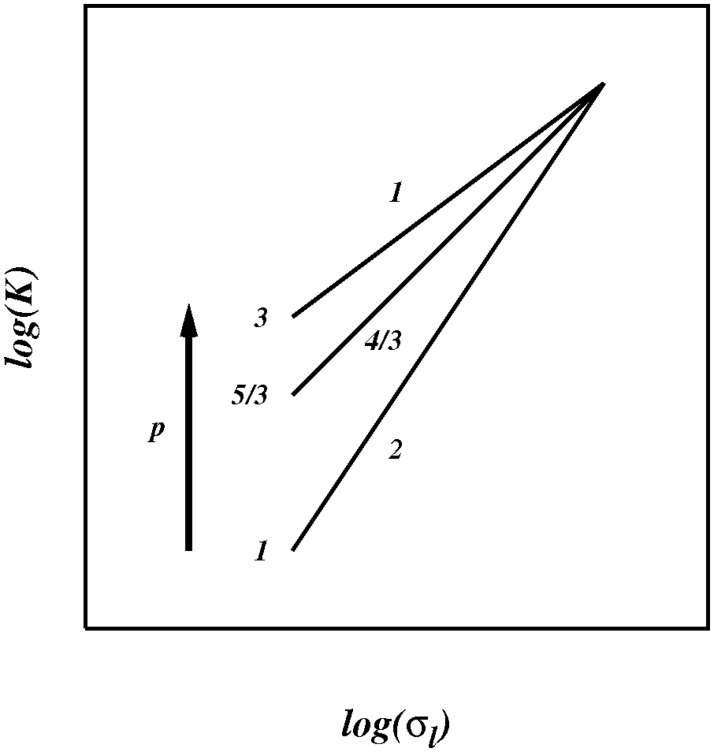
Sketch of the pair diffusion coefficient *K* against *p* for 1 < *p* ≤ 3, as predicted from the current theory for finite (short) inertial subranges, *R_k_* ≈ *C* ≪ ∞, (or *Re* ≪ ∞), Eq (49).

The non-local process, which is the first term in Eq (36), exists in the wavenumber range $\left[k_{1},k_{l}^{*}\right]$; but suppose that the inertial subrange itself is so small that it is close to the size of the locality kernel? Then the non-local range of scales would be absent to good approximation, and we might then observe a purely local diffusional process, as illustrated in the sketch in Fig 14.

However, due to the small size of the inertial subrange, the infra-red and ultra-violet corrections from the ends of the inertial subrange may have a relatively greater influence than in very large inertial subranges. So such a regime may only be approximately local in nature—i.e. a quasi-local regime,

$$\begin{array}{c} K(l,p)\approx \sigma_{l}^{(1+p)/2},\;\text{for}\;R_{k}\approx C \end{array}$$ (49)

**Fig 15:**
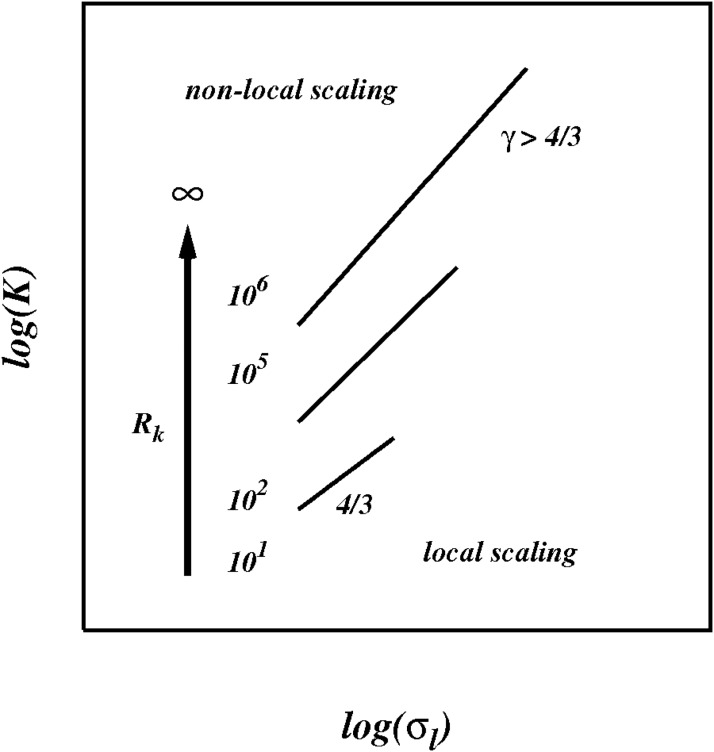
Sketch of the pair diffusion coefficient *K* against *σ*_*l*_, for Kolmogorov spectrum *p* = 5/3 as predicted from the current theory as the size of the inertial subrange increases from *R_k_* = 10 to *R_k_* → ∞.

As we progressively increase the size of the inertial subrange we would expect to see a smooth transition from the locality regime at moderate inertial subrange to the non-locality regime at very large (effectively infinite) subrange, as illustrated in the sketch in Fig 15.

Such a quasi-local regime if it exists is non-Richardson in character because locality was hypothesised for strictly infinite inertial subranges by Richardson.

### 5.4 Exposing the non-local process—Very small initial separation *l*_0_ ≪ *η*

We can ‘turn off’ the local process in Eq (36) by simply removing the ‘local’ part of the specturm—this is equivalent to taking a very small initial separation *l*_0_ ≪ *η*. Then there is a spectral gap between the *k*_0_ = 1/*l*_0_ and *k*_*η*_ ≪ *k*_0_ where *E*(*k*) = 0. If this gap is large enough then the spectrum between *k*_1_ ≤ *k* ≤ *k*_*η*_ will in efffect be non-local to the pair separation process so long as *σ*_*l*_(*t*) ≪ *η*.

Strictly speaking, this is not inertial range scaling any more because the separation is outside the inertial subrange, but because this system can be used to test the fundamental premise of the new theory, we will consider it here. According to the theory described in Eq (36), with the local range removed we should now observe purely strain dominated pair separation which has the diffusion coefficient scaling,

$$\begin{array}{c} K(l,p)∼\sigma_{l}^{2},\;\text{for}\;\sigma_{l}\ll \eta \end{array}$$ (50)

for all *p*. In fact, this scaling is independent of the size of the inertial subrange and also of the form of the spectrum, so long as *σ*_*l*_ ≪ *η*. Eq (50) is equivalent to exponential growth in time, *σ*_*l*_(*t*) ∼ *l*_0_ exp(*St*), where *S* depends upon the form of *E*(*k*).

But as *σ*_*l*_ approaches *η* from below, i.e. *σ*_*l*_/*η* → 1, the inertial subrange scaling will begin to take effect and we expect the scaling in (50) to change over.

## 6 Discussion

Richardson’s hypothesis of locality was based on the 1926 data-set of pair diffusion coefficients. However, the reappraised 1926 data presented here shows an unequivocal non-local scaling for the turbulent pair diffusivity, $K∼\sigma_{l}^{1.564}$, Fig 1. Consequently, the foundations of turbulent pair diffusion theory have been re-examined here in an effort to resolve one of the most important and enduring problems in turbulence.

A novel mathematical approach has been developed by expressing the pair diffusion coeffiicient through a Fourier integral decomposition. *a priori* assumptions regarding locality have *not* been made, and this has led to an expression for *K* as the sum of local and non-local contributions in Eq (36).

The main contribution of this investigation is to propose a new local-non-local theory of turbulent particle pair diffusion based upon the principle that the turbulent pair diffusion process in statistically stationary homogeneous turbulence is governed by both local and non-local diffusional processes. The theory preditcs the existance of two non-Richardson pair diffusion regimes in turbulence with generalized energy spectra, *E*(*k*) ∼ *k*^−*p*^.

For asymptotically infinite inertial subrange the pair diffusion coefficient scales like, $K\left(l,p\right)∼\sigma_{l}^{\gamma (p)}$, with (1 + *p*)/2 < *γ*(*p*) ≤ 2, in the range 1 < *p* ≤ 3, Eq (46), which is intermediate between the purely local and purely non-local scaling power laws. The reappraised 1926 geophysical data, Fig 1, provides support for the new theory in this limit.

For short inertial subrange quasi-local regimes are predicted in which the pair diffusion coefficient scales approximately locally like, $K\left(l,p\right)∼\sigma_{l}^{(1+p)/2}$, in the range 1 < *p* ≤ 3.

The theory also predicts that the existence of the local and non-local diffusional processes can be demonstrated in principle by islolating each one; first by taking short inertial subranges which would expose the local process, and then by taking very small initial separations which would expose the non-local process.

In summary, the new theory predicts the following two non-Richardson pair diffusion regimes:

$$\begin{array}{cc} local:K(l)=\sigma_{l}^{\gamma^{l}\left(p\right)}, & \gamma^{l}\left(p\right)=\left(1+p\right)/2,\;R_{k}/C\approx 1\;\left(Re\ll \infty \right) \end{array}$$ (51)

$$\begin{array}{cc} non-local:K(l)=\sigma_{l}^{\gamma (p)}, & \gamma^{l}\left(p\right)<\gamma \left(p\right)\leq 2,\;R_{k}/C\to \infty \;\left(Re\to \infty \right) \end{array}$$ (52)

The new theory could explain why DNS studies in pair diffusion have failed to observe non-local regimes of pair diffusion—because DNS would have to generate inertial subranges orders of magnitude bigger than currently possible. It was shown in Section 5.2 that in the locality limit, we should obtain the scalings $K\left(l\right)∼\sigma_{l}^{1.36}$ and 〈*l*^2^〉 ∼ *t*^3.125^ if we assume an intermittent spectrum of *p* = 1.72. However, no DNS study has so far reported a scaling greater than 〈*l*^2^〉 ∼ *t*^3^, which indicates that the size of the inertial subrange produced in DNS has not reached the minimum needed to observe locality scaling at the current time.

It is important to note that the theory presented here is based upon fundamental physical principles, and as such it is not dependent on any specific numerical method of simulation. However, the theory cannot predict quantitatively the power laws *γ*(*p*) themselves, or the precise size of the inertial subrange that will yield either of the two non-Richardson regimes. For these quantities, and in order to investigate other predictions of the new theory, ideally we would need experiments or DNS.

Neither laboratory experiments nor DNS can produce extended inertial subranges at the current time, which is an essential requirement in the present theory. However, some Lagrangian diffusion models can generate large inertial subranges. In a companaion paper, [27], such a numerical simulation method is used to investigate the new theory where all predictions of the new theory have been verified.
